# Factors associated with shunt dynamic in patients with cryptogenic stroke and patent foramen ovale: an observational cohort study

**DOI:** 10.1186/1471-2261-11-54

**Published:** 2011-08-26

**Authors:** Christian Tanislav, Maximilian Puille, Mathias Grebe, Nicole Sieweke, Jens Allendörfer, Wolfgang Pabst, Manfred Kaps, Frank Reichenberger

**Affiliations:** 1 Department of Neurology, Justus Liebig University Giessen, Am Steg 14, Giessen, 35392, Germany; 2 Department of Nuclear Medicine, Justus Liebig University Giessen, Friedrichstraße 25, 35392, Germany; 3 Department of Cardiology, Justus Liebig University Giessen, Klinkstraße 33, Giessen, 35392, Germany; 4 Department of Neurology, Neurologische Klinik Bad Salzhausen, Am Hasensprung 6, Nidda, 63667, Germany; 5 Institute for Biomedicine and Epidemiology, Justus Liebig University Giessen, Heinrich-Buff-Ring 44, Giessen, 35392, Germany; 6 Department of Respiratory Medicine, Justus Liebig University Giessen, Klinkstraße 33, Giessen, 35392, Germany

## Abstract

**Background:**

As previously reported there is evidence for a reduction in right to left shunt (RLS) in stroke patients with patent foramen ovale (PFO). This occurs predominantly in patients with cryptogenic stroke (CS). We therefore analysed factors associated with a shunt reduction on follow-up in stroke patients suffering of CS.

**Methods:**

On index event PFO and RLS were proven by transesophageal echocardiography and contrast-enhanced transcranial Doppler-sonography (ce-TCD). Silent PE was proved by ventilation perfusion scintigraphy (V/Q) within the stroke work-up on index event; all scans were re-evaluated in a blinded manner by two experts. The RLS was re-assessed on follow-up by ce-TCD. A reduction in shunt volume was defined as a difference of ≥20 microembolic signals (MES) or the lack of evidence of RLS on follow-up. For subsequent analyses patients with CS were considered; parameters such as deep vein thrombosis (DVT) and silent pulmonary embolism (PE) were analysed.

**Results:**

In 39 PFO patients suffering of a CS the RLS was re-assessed on follow-up. In all patients (n = 39) with CS a V/Q was performed; the median age was 40 years, 24 (61.5%) patients were female. In 27 patients a reduction in RLS was evident. Silent PE was evident in 18/39 patients (46.2%). Factors such as atrial septum aneurysm, DVT or even silent PE were not associated with RLS dynamics. A greater time delay from index event to follow-up assessment was associated with a decrease in shunt volume (median 12 vs. 6 months, *p *= 0.013).

**Conclusions:**

In patients with CS a reduction in RLS is not associated with the presence of a venous embolic event such as DVT or silent PE. A greater time delay between the initial and the follow-up investigation increases the likelihood for the detection of a reduction in RLS.

## Background

As previously reported there is evidence for a reduction in right to left shunt (RLS) or even a functional closure of a patent foramen ovale (PFO) on follow-up with predominance in patients with cryptogenic stroke (CS) [1]. As a functional PFO closure was observed after treatment of acute massive pulmonary emboli, a fluctuation in pulmonary atrial pressure might be involved in the mechanism of shunt dynamic across a PFO [2-4]. Considering that silent pulmonary embolism (PE) is a frequent finding in patients with CS and PFO, it raises the question whether an increase in right atrial pressure due to silent pulmonary embolism may have a similar impact on the development of RLS dynamics [5]. We therefore investigated the relevance of silent PE in a cohort of PFO patients with CS with a re-assessment of RLS on follow-up.

## Methods

### Patients

The eligibility for the study entry was proved by reviewing case files from consecutive patients. The inclusion criteria were as follows: stroke or transient ischemic attack (TIA), RLS and PFO proven by transesophageal echocardiography (TEE) and contrast-enhanced transcranial Doppler/duplex ultrasound (ce-TCD), past index event at least one month prior to study entry. After obtaining the written informed consent the RLS was re-assessed by ce-TCD. Among 130 eligible patients, 39 patients suffering of CS were re-assessed with respect to shunt persistence. For categorize the ischemic stroke subtypes criteria established in the Trial of Org 10172 in Acute Stroke Treatment (TOAST) were used [6]; in contrast to determined etiologies (large-artery atherosclerosis, cardioembolism, small-vessel occlusion, and strokes of other determined etiologies) in patients with strokes of undetermined etiology (also termed as cryptogenic strokes) the PFO and the paradoxical embolism represented the single cause of stroke which comes into consideration.

Baseline demographic and clinical information were derived from a stroke registry in which the patients had been included prospectively, and additionally from case notes recorded at the index event (T0) [7]. On follow-up assessment (T1) the following information was recorded systematically: treatment administrated during follow-up period and time interval between index event and follow-up visit (T0-T1) in months.

The study protocol war reviewed and approved by the ethical committee of the Justus Liebig University Giessen.

### ce-TCD

A 2 MHz probe (Philips HP SONOS 5500, Philips Healthcare, Hamburg, Germany) was used to carry out the ce-TCD. The contrast agent, based on a D-galactose microparticle solution (Echovist™, Bayer Vital, Berlin, Germany), was prepared according to the manufacturer's instructions, and injected into a large cubital vein of the left arm. The contrast agent was administered as two separate boluses of 5 ml each, one while normal respiration and the other while a 10 seconds Valsalva strain. The Valsalva manoeuvre was practiced with the patient before the procedure. The examination was performed standardized according to the recommendations of the international consensus meeting [8]. A curtain pattern was considered when an individual MES count was not possible.

On follow-up the examiner carrying out the ce-TCD did not have access to clinical data and initial measurements. All examinations (T0 and T1) were evaluated offline by two experts in a standardized protocol, blinded from any clinical data and the individual chronological order. In case evaluations were graded differently (different MES count), a consensus read was undertaken. A change in shunt volume was defined as a difference of ≥20 microembolic signals (MES) or no evidence of RLS on follow-up examination.

### TEE

TEE was performed by experienced echocardiographers from the Department of Cardiology using a 4-7 MHz multi-plane probe (HP Sonos 5500, Philips Healthcare, Hamburg, Germany). The same examiner carried out 95% of the TEEs.

To detect an intracardiac shunt, 10 ml of the contrast agent (Echovist™, Bayer Vital, Berlin, Germany) was administered by bolus injection into a large antecubital vein. RLS was evident if the transit of microbubbles from the right to the left atrium occurred spontaneously or during a subsequent Valsalva strain. PFO was diagnosed when at least three microbubbles were detected in the left atrium within three heart beats after appearance in the right atrium. An atrial septal aneurysm (ASA) was diagnosed when the atrial septum extended at least 11 mm into the left or the right atrium, or both. An excursion of minimum 5 mm of the septum primum into either the left or right atrium with respect to a perpendicular line to the fossa ovalis plane was considered as hypermobile atrial septum (HAS).

### Ventilation/perfusion scintigraphy (V/Q)

In patients with CS and proven PFO a V/Q was undertaken while hospitalisation for the index event as part of the routine workup. The radioaerosol delivery system VENTICIS II™ was used for nebulizing a solution of Tc99m-DTPA. For perfusion scintigraphy 90 MBq of Tc99 m labelled albumine macroaggregates were slowly injected intravenously during 3-4 breath cycles. Image acquisition was performed with a Siemens MultiSpect III gamma camera. Projection data were scatter corrected and reconstructed as for single photon emission tomography (SPECT) using an iterative reconstruction algorithm. A perfusion defect is visualized indirectly by perfusion scanning as a parenchymal defect related to the embolized artery, and is confirmed by combined ventilation scanning, showing a normal regional ventilation.

**Figure 1:**
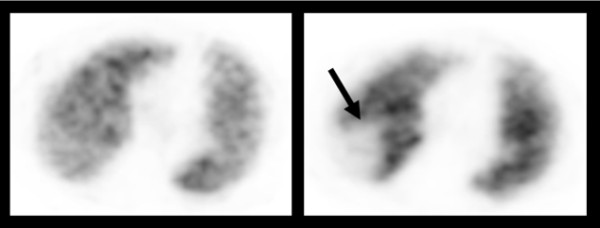
**Perfusion defect detected by V/Q (SPECT reconstruction)**. Regular ventilation left, perfusion defect right (arrow).

Blinded from clinical data all scans were independently evaluated by two experts according to the PIOPED (prospective investigation of pulmonary embolism diagnosis) criteria modified for SPECT images, as described by Reinartz et al [5,9,10]. In scans graded differently a consensus read was undertaken. Scans showing at least subsegmental perfusion defects with corresponding regular ventilation were considered consistent with PE (Figure 1).

### Statistical analysis

For analysing factors associated with shunt volume dynamics in patients suffering of CS non-parametric data were compared using the Mann-Whitney *U*-test; comparisons of dichotomized data were performed by Fisher's exact test.

## Results

Among 39 patients suffering of CS a reduction in shunt volume was evident in 27 patients (69.2%). In these patients (n = 39) the median age was 40 years, 24 patients (61.5%) were female. Silent PE (Figure 1) was demonstrated in 18 patients (46.2%), whereas a DVT was found only in 3 patients (7.7%). An ASA was found in 11 patients (28.2%) and in 10 patients (25.6%) a HAS was evident. Factors such as ASA, DVT or silent PE were not associated with a RLS reduction over time (Table 1). A greater time delay from index event to follow-up assessment was associated with a decrease in shunt volume (median 12 vs. 6 months, *p *= 0.013).

**Table 1:** Factors associated with PFO shunt dynamic in patients with cryptogenic stroke and PFO

	Total patient cohort n = 39 (%)	RLS volume change n = 27(%)	No RLS volume change n = 12 (%)	*P*
Age (years) median (range)	40.0 (19.7 - 65.2)	42.8 (21.4 - 65.2)	38.1 (19.7 - 64.3)	0.58
Sex				0.47
Female	24 (61.5)	16 (59.3)	8 (66.7)	
Male	15 (38.5)	11 (40.7)	4 (33.3)	
Use of anticoagulants	28 (71.8)	20 (74.1)	8 (66.7)	0.46
ASA	11 (28.2)	8 (29.6)	3 (25.0)	0.54
HAS	10 (25.6)	6 (22.2)	4 (33.3)	0.36
DVT	3 (7.7)	1 (3.7)	2 (16.7)	0.22
Silent PE	18 (46.2)	12 (44.4)	6 (50.0)	0.51
T0-T1 (months) median/mean (SD)	12 (6 - 60)	12 (7 - 60)	6 (6 - 25)	0.013

RLS, right to left shunt; ASA, atrial septal aneurysm; HAS, hypermobile atrial septum; DVT, deep vein thrombosis; PE, pulmonary embolism; T0-T1 time delay index event to follow-up assessment.

In the total cohort of 102 patients investigated with respect to shunt persistence in 63 patients other causes for stroke were evident. In these patients a reduction in RLS was less frequently observed (4/63 vs. 29/39, *p *< 0.001). Patients with CS were younger (median 40 vs. 63 years, *p *< 0.001) and had more frequently oral anticoagulants for the secondary stroke prevention (28/39 vs. 12/63, *p *< 0.001) than patients with other determined stroke aetiologies.

## Discussion

In this specific group of patients with cryptogenic stroke and PFO factors such as the use of oral anticoagulants for secondary prevention, an associated ASA or silent PE diagnosed within the stroke work-up on index event were not associated with a decrease in RLS shunt volume across a PFO. The time delay from index event to the follow-up assessment was proven the single factor determining a decrease in RLS across a PFO, suggesting the later the follow-up assessment the greater the likelihood for a RLS reduction. However, due to the study design, there are limitations regarding a precise determination of a time interval.

A functional PFO opening/closure was observed during the course and treatment of an acute massive PE, suggesting that a pressure fluctuation within the right atrium might be responsible for this phenomenon [2,3]. Yet it far remains uncertain, whether also a moderate pressure increase in pulmonary arterial pressure as induced by silent PE is sufficient to facilitate a change in RLS across a PFO [11,12]. Considering the high proportion of patients with silent PE among patients with CS this mechanism appears likely [5]. Despite the plausibility of such an interrelation, clear evidence that silent PE is involved in the mechanism of shunt dynamic could not be derived from our data. However, a definitive conclusion for or against this mechanism can yet not be drawn, especially considering the ambiguity, whether the perfusion defects detected in our study reflect recent events; the morphological analysis of subsegmental defects in the V/Q does not allow a precise determination of the date of onset and all patients were asymptomatic for PE [5]. A direct involvement in the pathophysiology of paradoxical embolism and shunt dynamic remains uncertain. Thus, this limitation needs to be taken into account when interpreting our results.

Verifying our results appears mandatory as they could be of immediate relevance. Cuppini et al. found silent PE in a high percentage of patients admitted with acute DVT [13]. In the majority of cases perfusion defects resolved within ten days after anticoagulant treatment. This suggests high efficacy of an anticoagulant treatment [13]. Assuming an association between silent PE and shunt dynamic, an effective treatment of PE could favourably influence a shunt dynamic: it potentially may lead to a shunt reduction or even a functional closure.

However, further investigations are of high priority as a better understanding of the pathophysiology behind the shunt dynamic across a PFO is of relevance for the development of new individualized treatment strategies. In particular, the spontaneous and functional closure of a PFO would be of major clinical importance, potentially facilitating an individualised decision for or against an invasive intervention. Furthermore circumstances which potential impact on the maintenance of a functionally "closed" PFO as well as factors involved in the "opening" of a PFO would be of particular interest as they could be considered when making recommendations.

The main limitation of the present study is the small number of patients. However, it represents a unique analysis in patients with CS and a re-assessment of RLS on follow-up in association with silent PE. Even though collected retrospectively, all V/Q scans were independently reassessed by two experts, who were blinded from any clinical data [5].

## Conclusions

In patients with CS a reduction in RLS is not associated with the presence of a venous embolic event such as DVT or silent PE. A greater time delay for the follow-up event increases the likelihood for proving a reduction in RLS on follow-up. A verification of these results seems mandatory, as they might implicate a high clinical relevance. A better understanding of the mechanism of PFO shunt dynamic potentially facilitates new treatment strategies in the primary as well as in the secondary prevention. Especially the identification of further factors which maintain a functionally "closed" PFO and particularly of those factors involved in an "opening" mechanism would be of major interest.

## Abbreviations

PFO: patent foramen ovale; RLS: right to left shunt; CS: cryptogenic stroke; T0: time at index event; T1: time at follow-up assessment; T0-T1: time interval between the index event and follow-up assessment; ce-TCD: contrast-enhanced transcranial Doppler/duplex ultrasound; TEE: transesophageal echocardiography; ASA: atrial septum aneurysm; HAS: hypermobile atrial septum; PE: pulmonary embolism; DVT: deep vein thrombosis; MES: microembolic signals; PIOPED: Prospective Investigation Of Pulmonary Embolism Diagnosis.

## Competing interests

The authors declare that they have no competing interests.

## Authors' contributions

CT, JA, MP and MG carried out the data collection and drafted the manuscript. CT and JA participated in conception and design. WP and CT performed the statistical analyses. All authors were involved in the analysis and interpretation of the results. All authors revised the manuscript critically for important intellectual content and were involved in drafting the manuscript. All authors read and approved the final version of the manuscript.

## Pre-publication history

The pre-publication history for this paper can be accessed here:

http://www.biomedcentral.com/1471-2261/11/54/prepub
